# Shame and disgust in patients with inflammatory skin diseases: a systematic review of psychological correlates and psychotherapeutic approaches

**DOI:** 10.3389/fmed.2025.1620940

**Published:** 2025-06-30

**Authors:** Jakob Fink-Lamotte, Sebastian Wehle, Frederica Brinkmann, Marie Pelzer, Cornelia Exner, Christian Stierle

**Affiliations:** ^1^Clinical Psychology, University of Potsdam, Potsdam, Germany; ^2^Clinical Psychology and Psychotherapy, University of Leipzig, Leipzig, Germany; ^3^Experimental Psychology and Methods, University of Leipzig, Leipzig, Germany; ^4^School of Psychology, Fresenius Hochschule für Wirtschaft und Medien, Hamburg, Germany; ^5^Health Psychology and Paedagogy, Riga Stradins University, Riga, Latvia

**Keywords:** acne, compassion, disgust, psoriasis, shame, atopic eczema

## Abstract

**Introduction:**

Skin diseases are among the most common diseases worldwide and can cause severe psychological and social impairments. Negative self-directed emotions like shame and disgust may be important in the development and progression of these diseases, and thus, patients may benefit from psychotherapeutic approaches targeting shame and self-disgust. The first aim of this systematic review is to investigate the existing literature regarding shame and disgust as psychological correlates of inflammatory skin diseases. The second aim is to review the existing literature concerning the evidence of the efficacy of mindfulness-based and compassion-based therapy for alleviating shame and self-disgust in the context of skin diseases.

**Methods:**

Therefore, we carried out a systematic literature review via the databases PubMed, Web of Science and PSYINDEX.

**Results:**

46 manuscripts were included in this review. Research shows that acne vulgaris, psoriasis, and atopic eczema are accompanied by a severe psychosocial burden, shame, and self-disgust, often due to highly visible skin lesions in affected patients. The use of mindfulness-based and compassion-based approaches is already being studied to address the experiences of shame and disgust due to these diseases, and initial promising results indicate that they can be considered beneficial in the holistic therapy of skin diseases.

**Discussion:**

This systematic review shows that skin disorders have a significant psychosocial impact, leading to shame and self-disgust, especially due to the manifestation of visible skin lesions in affected patients. Mindfulness- and compassion-based approaches are currently being studied as potential treatments for the psychosocial impacts of skin diseases, and show promising results in addressing affected patients’ psychological burden.

## Highlights

1. Patients with the three included inflammatory skin diseases are burdened by shame and self-disgust, often related to experiences and fear of social rejection
2. Shame and self-disgust may lead to social withdrawal, affecting life quality, treatment compliance and symptom progression, but causal evidence is lacking
3. Mindfulness- and compassion-based approaches are promising in alleviating shame and self-disgust

## 1 Introduction

Skin diseases, including fungal skin diseases, other skin and subcutaneous conditions, and acne, ranked among the top ten most common diseases worldwide in 2010 (1). In a study involving 90,880 employees conducted from 2004 to 2009, 3.9% had acne vulgaris, 2% had psoriasis, and 1.3% had atopic eczema (2), making these inflammatory dermatological conditions three of the most prevalent skin diseases. A British report revealed that 14% of surveyed dermatological patients reported that their skin conditions were exacerbated by psychological factors, while 85% noted that the interference with their social relationships was the most distressing aspect of their illness (3). Importantly, the study highlighted a notably higher suicide rate among patients with inflammatory skin diseases, surpassing that in the general population (4). 17% of surveyed dermatological patients required psychotherapeutic treatment (3).

### 1.1 The emotional consequences of skin diseases: disgust and shame

**FIGURE 1:**
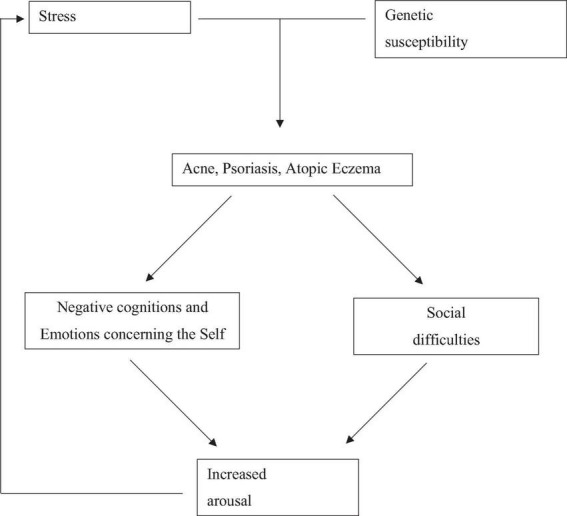
The adapted biopsychosocial model of skin disease development and progression, adopted from Kellett and Gilbert (7).

Rook and Wilkinson (5) already argued in the late 1970s that “*[.] the role of emotional factors on diseases of the skin is of such significance that, if they are ignored, the effective management of at least 40% of the patients attending departments of dermatology is impossible*” (6). From an evolutionary standpoint, Kellett and Gilbert (7) center their focus on self-related negative emotions like shame and disgust. Their bio-psycho-social model, as depicted in Figure 1, serves as the foundation for this systematic review. They argue that acne’s development and progression stem from a combination of genetic factors and the stressors of puberty. The authors propose that when acne symptoms become prominent, individuals experience negative thoughts and emotions, especially related to the self, along with social challenges. These internal struggles and interpersonal issues adversely affect mood, behavior, and immune system function, further impacting stress levels, acne symptoms, and the emergence of additional psychological problems. The bio-psycho-social model of acne (Figure 1) can also be applied to the clinical presentation of psoriasis and atopic eczema, as all three conditions involve inflammatory skin issues that can negatively impact social life, emotional and psychological wellbeing. The connection of stress, social factors, and negative emotions with skin diseases is supported by evidence (6, 8), while a systematic review on shame and self-disgust in skin diseases is lacking.

The basic human emotion of *disgust* is described as a form of rejection that arises from the need to distance oneself from a contaminated stimulus and is characterized by feelings of nausea and revulsion (9). Disgust is considered to be an adaptive emotion, which is an evolved response to objects in the environment that pose a (perceived) threat in terms of contagion through infectious diseases (10). *Self-disgust* describes feelings of reluctance and repulsion directed against specific aspects of one’s own person (11), whereas (*general) shame* is believed to be an incapacitating emotion that is accompanied by the feeling of being small, inferior, and of “shrinking,” whereby the self, as a whole, is devalued and considered to be inadequate, incompetent, and worthless (12). Shame is viewed from a more differentiated perspective, and a distinction is made between external and internal shame. *External shame* refers to the experience of the self as existing negatively in the minds of others, and thus as having visible deficits, failures, or mistakes (13). *Internal shame*, by contrast, is linked to the inner dynamics of the self and one’s judgements and evaluations (13). The distinction between shame and *embarrassment* is debated, with researchers suggesting distinctions in terms of intensity of affect, severity of transgression or patterns of attribution to the *presented* (embarrassment) vs. the *core* (shame) self (14). However, despite empirical evidence for their distinctness (15, 16), shame and embarrassment are not always distinguished unequivocally, and, as a result, when many researchers speak of embarrassment, it is more accurate to think of shame (17).

Shame in the context of skin disease may arise from the experience of stigma (18). Stigma refers both to social reactions to attributes seen as degrading or devaluating (e.g., blemished skin), and to the internalization of such devaluation, termed self-stigma (19). Self-stigma and shame are thus closely related, both involving the devaluation of self, and are sometimes used interchangeably (20, 21). The impacts of stigma in skin diseases have been reviewed elsewhere (18, 22), whereas shame and self-stigma have not been investigated separately in skin diseases.

Kellett and Gilbert (7) also explore the impact of social difficulties and the importance of attractiveness on body shame reactions. In the context of body shame reactions, Gilbert (13) defines it as shame related to one’s own body, particularly concerning skin. In society, clear skin is idealized as a feature of “desirable individuals” (p. 10), and blemished or diseased skin can be perceived as a sign of poor overall health. Consequently, those affected may fear eliciting negative emotions like fear, anger, or disgust in others, potentially diminishing their desirability in various aspects, notably sexually.

Despite the importance of shame and self-disgust for the connections between social challenges, wellbeing, and symptom exacerbation posited in the bio-psycho-social model, the influence of these emotions on the wellbeing and treatment of individuals with skin disorders has received limited attention. Thus, this paper’s primary goal is to review existing literature, specifically focusing on the evidence supporting shame and self-disgust as psychological consequences and potential enhancers of inflammatory skin diseases. Reviewing the evidence will help identify gaps in the literature and guide further research into the psychological burdens and treatment of patients with skin diseases.

### 1.2 Psychotherapy for dermatological diseases

In various studies with patients suffering from atopic eczema or psoriasis vulgaris, the beneficial effects of combined dermatological and psychosocial interventions on the improvement of the skin condition, reduction of scratching frequency, and psychosocial parameters have been demonstrated (23). Good results are also obtained in approaches like relaxation, breathing, and art therapy, or psychodynamic catathymic image perception (24). Cognitive therapies focus on the dysfunctional appraisal of stressful events (25). Most approaches, however, lack a focus on challenging emotions such as shame and self-disgust (23). Modern psychotherapy has increasingly embraced mindfulness techniques, derived from Buddhism, such as mindfulness-based stress reduction (MBSR) and mindfulness-based therapies (MBT). These approaches adapt mindfulness from Buddhism to promote acceptance of current situations, reduce mental distress, and enhance overall psychological wellbeing. Stress-related skin conditions can benefit from these mindfulness practices, as they help reduce the mental noise and foster wisdom, ultimately increasing tolerance to stress and improving skin health (26). Additionally, self-compassion, a key concept in compassion-focused therapy (CFT), involves viewing oneself kindly and empathetically during challenging times, recognizing the universality of suffering, and mindfully accepting it (27). Unlike shame, which entails self-devaluation and a harsh self-critical relationship, self-compassion nurtures a loving, understanding, and forgiving self-relationship (28). By promoting better self-regulation and motives to alleviate suffering, mindfulness and self-compassion interventions can effectively address pathological shame and self-disgust in the context of skin diseases. As such, the second objective of this study is to review existing literature on the scientific evidence for using compassion-based or mindfulness-based therapies to address self-disgust and shame in individuals with skin diseases.

## 2 Materials and methods

We chose a methodological approach based on the PRISMA statement (29) for study selection, systematic search and data synthesis. Ethics approval is not required for this type of research at our institution.

### 2.1 Study selection

Studies were selected using the inclusion and exclusion criteria defined prior to the literature search. Inclusion criterion 1 included studies investigating emotions of shame (or self-stigmatization or embarrassment) or self-disgust or experience of disgust in the context of (psycho-)dermatological diseases (psoriasis, acne, atopic eczema). Criterion 2 included studies investigating mindfulness-based or compassion-focused-therapy regarding the emotions shame or self-disgust in patients with dermatological diseases (psoriasis, acne, atopic eczema). We did not define any excluding design parameters and only included studies focusing on adult populations (≥ 18 year).

Furthermore, we excluded pharmaceutical studies, quality assessments of clinical instruments or questionnaires, studies linking other types of emotions (e.g., nausea or anxiety) to skin diseases, and studies focusing on other types of dermatological diseases (e.g., sexually infectious diseases or skin picking). Additionally, as the effects of mindfulness interventions for improving quality of life (30) and experience of stigma in patients with skin diseases (18, 22) have recently been reviewed, studies with a primary focus on quality of life or stigmatization (without differentiating aspects of stigma) in the context of dermatological diseases were excluded. Studies examining mindfulness or self-compassion as traits or variables in therapy research were also excluded, a comprehensive review for the context of dermatological diseases was recently published (31). Studies examining compassion or mindfulness strategies outside of the context of dermatological diseases, or emotions of shame or disgust, were also excluded.

### 2.2 Search strategy

Based on our criteria, search terms were defined and combined logically to build a search operator. We used wildcards to include multiple concrete forms of the same word-stems: [(skin disease) OR (skin condition*) OR (disease risk) OR (dermatolog*) OR (acne) OR (eczema) OR (psoriasis)] AND {(shame) OR (disgust) OR (mindfulness) OR (compassion) OR (self-compassion) OR (bodily suffering) OR [(emotion*) AND (psycho*) AND (psychotherap*)] OR [(emotion*) AND (psycho*) AND (dermatolog*)]}. PubMed provides an option to add a NOT-operator. This was added for the following terms: NOT sexual abuse[Title/Abstract] NOT childhood[Title/Abstract] NOT dement*[Title/Abstract] NOT HIV/AIDS[Title/Abstract] NOT HIV[Title/Abstract] NOT breast cancer[Title]. The search results were combined using reference management software.

The systematic search was conducted in January 2022 via the databases PubMed, Web of Science and PSYINDEX and yielded *N* = 6,505 studies (*n* = 2,097 Doublettes). One study [(32), retrieved via Google Scholar] was published later in 2022 and was subsequently added to the search results. One relevant study (32) was excluded by our search operator because it contained the word “childhood” in the abstract.

### 2.3 Data synthesis

**FIGURE 2:**
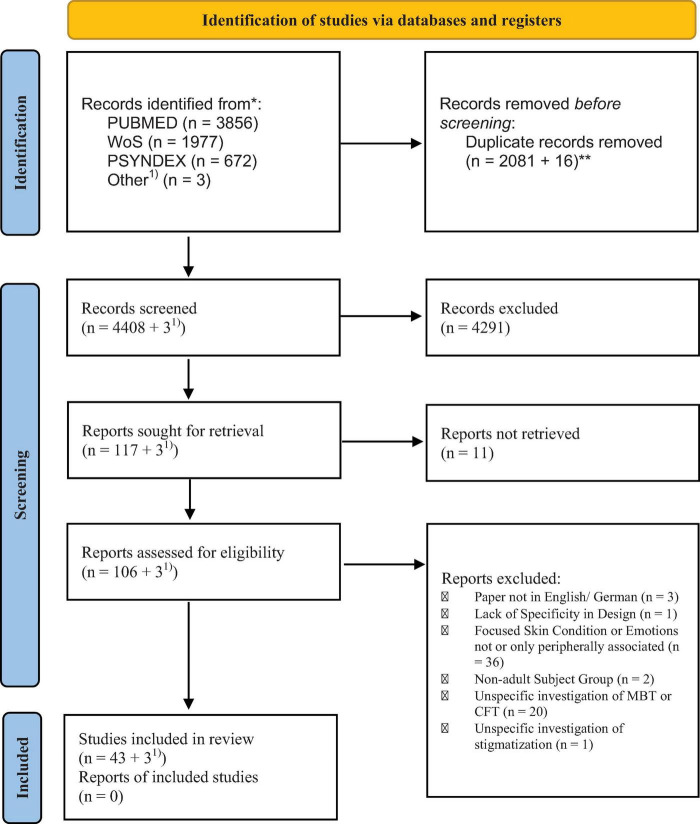
PRISMA flow chart. ^1^One Author reported a google-scholar-based recommendation for a study that had not been in the original screening but passed inclusion criteria. Reviewers recommended another two relevant studies. *Consider, if feasible to do so, reporting the number of records identified from each database or register searched (rather than the total number across all databases/registers). **If automation tools were used, indicate how many records were excluded by a human and how many were excluded by automation tools. *** by automation tool + by human during screening [From (29)]. For more information, visit: http://www.prisma-statement.org.

The screening of the results was conducted between January and September 2022 (see Figure 2 for the PRISMA flow chart). A random selection of 496 studies was screened independently by all three reviewers based on the inclusion/exclusion criteria outlined above. The remaining studies were split equally and randomly between the three reviewers and screened accordingly. The set of randomly selected studies was used to estimate the inter-rater reliability. Although all three reviewers agreed concerning a binary decision (in-/exclusion) for 96.6% of the studies, the inter-rater reliability of the study selection was weak (k_fleiss_ = 0.359). Disagreements regarding the selection existed in the case of *n* = 17 studies and were discussed by all co-authors. Further inspection showed that two studies with relation to the quality of life also contained aspects of the emotion of embarrassment (33, 34) and were thus considered relevant. Furthermore, the inclusion and exclusion criteria related to social anxiety [included; (35, 36)], feelings of stigma [included; (35, 37, 38)], and skin picking [excluded; (39)] were interpreted differently by the reviewers. After discussing and resolving the disagreements, the agreement of study selection was 97.5% with substantial inter-rater reliability (k_fleiss_ = 0.659). One study (40) was falsely excluded in the screening and included in the review process thanks to one reviewer’s comment. All studies for which reviewers were not able to resolve their disagreements were included in the next step of retrieving the full manuscripts.

The full manuscripts of the remaining studies were subsequently retrieved. Studies were excluded if the full text was not available (*n* = 11), not written in English or German (*n* = 3), had a focus on skin disease or emotion that did not fit the inclusion criteria (*n* = 36), did not include an adult sample (*n* = 2), or investigated mindfulness and self-compassion as traits or not in relation to disgust or shame (*n* = 21). The main results across each group of studies were then summarized for a synthesis of the main outcomes.

### 2.4 Risk of bias assessment

The risk of bias was assessed independently using the NHLBI quality assessment tool (41). The tool consists of a catalog of questions that ask key questions about the internal validity of the respective study. There is a separate, suitable list of questions for each study type (controlled intervention studies, systematic reviews and Meta-Analyses, Observational Cohort, Case-Control Studies, etc.). Each point is queried with yes/no questions (unsuitable or non-answerable questions are answered with NA or “not reported”) and categorized as “good,” “fair” or “poor” (no quantification) according to predefined rules. Five independent reviewers rated the studies according to the applicable criteria. The detailed results of the risk of bias assessment can be found in the Supplementary Table 1. Systematic Reviews and Meta-Analyses often exhibited poor quality, raising concerns about the reliability of their findings. Controlled Intervention Studies, on the other hand, presented a heterogeneous picture ranging from poor to good quality, indicating the need for more standardized methodologies. Most studies fell within the category of Observational Cohort and Cross-Sectional Studies. These studies demonstrated varying degrees of quality, generally falling within the fair to good range. Additionally, a selection of qualitative studies was also included in the analysis contributing to the diversity of the evidence base. Based on the COREQ-Assessment (42) they showed fair to good quality.

## 3 Results

### 3.1 Shame, embarrassment, and self-stigma in dermatological diseases

Most of the literature that has examined shame, embarrassment, and self-stigma in dermatological conditions and that was included in the review comprised studies that examined patients with psoriasis. A total of 33 out of 46 included studies (72%) focussed exclusively or i.a., on psoriasis, while fewer studies focussing on acne (9 studies, 19%) or atopic eczema (4 studies 8.7%) were found.

Several questionnaire studies show that people with skin diseases displayed high levels of shame (17, 43–46), albeit not general shame (47) [see review by (48)]; skin shame (48); social shame (49). In a cross-sectional study with 166 psoriasis patients, Jankowiak et al. (50) showed that higher levels of shame were especially found in cases with visible skin lesions [see review by (51)] and that older patients reported less shame.

In addition, questionnaire studies found high levels of embarrassment (17, 33, 43, 45, 46, 52–55) associated with high self-consciousness about the skin disease (17, 56, 57), as well as high levels of self-stigma (17, 34, 58) in people with skin diseases. Again, visible lesions (e.g., scars) were associated with more embarrassment (59), and in psoriasis patients, this was even more acute than in people suffering from atopic eczema (60). Additionally, in a meta-analysis of ten qualitative studies of people with various dermatological conditions, emotional experience with embarrassment and shame emerged as one of the main themes (61). In one study, shame experience was also highlighted as the unifying factor between psoriasis and acne inversa patients in particular (62).

Lahousen et al. (32) found that patients with psoriasis reported significantly higher levels of skin-related shame and disgust than healthy controls, as well as more negative appraisals of self-touching and parental touching. Interestingly, severity of skin condition was unrelated to shame and disgust. A qualitative study also found that people suffering from skin diseases had an impaired self-image and low self-esteem (17, 63, 64). In a study on sexual problems in psoriasis, the majority of men reported feeling embarrassed and less attractive due to skin lesions, and at least occasionally ashamed with sexual partners (65). Psoriasis patients often cited feelings of being stared at, and others’ erroneous beliefs that the disease was contagious, as stressors (65, 66). These feelings may cause avoidant behavior, thus perpetuating social exclusion and explaining the link between shame, stigmatization, and depression as well as lower quality of life (67).

The study by Ginsburg and Link (37) showed that the fear of social exclusion harbored by psoriasis patients is not completely unjustified: 19% of patients experienced episodes of gross rejection as a result of their psoriasis. These experiences were accompanied by feelings of stigmatization. Magin et al. (68) also found embarrassment and low self-esteem as a result of teasing and bullying in people with dermatological conditions while Schielein et al. (69) showed that the main reason affected patients avoided sexual activity was shame and fear of rejection [see also (70)]. Since these feelings are fundamentally associated with social withdrawal and depression, Vladut and Kállay (71) call for multidisciplinary treatment in their review.

In connection with psoriasis, Ginsburg and Link (38) worked out six dimensions of self-stigma via a questionnaire study: the anticipation of rejection, a feeling of being flawed, sensitivity to others’ attitudes, guilt and shame, secretiveness, and positive attitudes. The authors suggested that a high experience of stigma might be associated with increased non-compliance with treatment and thus symptom exacerbation.

In a study by George et al. (72), what patients found helpful in dealing with the fear of social exclusion was active listening, shared decision-making, and communication of hope.

### 3.2 Self-disgust and experience of disgust in dermatological diseases

The state of research on disgust in dermatological diseases is considerably less comprehensive than that on shame and embarrassment. Mento et al. (73) also came to this conclusion in a literature review, according to which anger and disgust are neglected in studies. In a qualitative study, Wahl et al. (74) show that patients with psoriasis describe their bodies (especially in the case of visible skin rashes) as “offensive” and regard themselves as “unclean, infectious, disgusting, leprous, ugly, unattractive, strange, big, or different.” [see also (67)]. In an ethnographic qualitative study in different countries, dermatological patients described themselves with disgust and self-loathing (75).

In a questionnaire study, over 60% of patients with psoriasis reported experiencing strong self-disgust (76), and the findings showed that sex, age, depression, and perceived stigmatization were predictors of self-disgust whereby the relationship between stigmatization and depression was mediated by self-disgust. Moreover, there is evidence that dermatological patients experience disgust in others differently from skin-healthy people. In an approach-avoidance task, patients with psoriasis and their significant others more strongly avoided faces displaying disgust, but not other emotions, compared to controls (40). In an fMRI study by Kleyn et al. (77), psoriasis patients showed a reduced ability to recall faces with disgust reactions compared to controls. The authors concluded that this related to learned coping mechanisms to protect themselves from the reaction of disgust in others.

### 3.3 Psychotherapy for dermatological diseases: compassion-based-therapeutic approaches

Although few studies to date have examined the effects of compassion-focused therapy on shame or disgust in skin conditions, empirical evidence supports the potential of CFT for reducing shame and enhancing treatment for patients with skin diseases. In a two-week randomized controlled trial (RCT) on 75 patients with facial acne who experienced skin-related distress and varying levels of depression, Kelly et al. (78) found that two compassion-focused interventions significantly reduced shame, as well as skin discomfort and depression, in comparison to a passive control. More mixed results were found in a four-week compassion focused self-help program (79). In this single-arm feasibility study with a small sample, two patients with initially high levels of shame and self-criticism reported significant reductions of shame and self-criticism at the end of the program, while two patients with relatively lower baseline levels experienced significantly increased levels of shame following the intervention (79). Thus, the limited evidence mostly supports the potential of CFT as an approach for individuals with skin diseases, particularly in reducing shame, self-criticism, and psychological distress.

### 3.4 Psychotherapy for dermatological diseases: mindfulness-based therapeutic approaches

For the effects of mindfulness-based approaches on shame and disgust in the context of skin disease, our search yielded only one study comparing MBT against CFT. In a feasibility RCT, Muftin et al. (80) tested a mindfulness self-help online program against a CFT program in 130 patients with psoriasis. Results showed that both four-week programs were acceptable and helpful in reducing shame and improving quality of life, suggesting that MBT may have comparable potential to CFT in alleviating shame and distress in patients with skin conditions.

## 4 Discussion

The primary goal of this systematic review was to search for evidence supporting that shame and self-disgust are psychological correlates of inflammatory skin diseases. The results provide strong evidence for shame, embarrassment, and self-stigma as significant aspects in dermatological conditions, particularly in individuals with psoriasis. In this line, patients often suffer from high emotional stress and social exclusion. Visible skin lesions increase the experience of shame, although older patients are often less affected. Shame and self-stigmatization do not necessarily correlate with the severity of the disease, but rather with the awareness of the skin problems and the social reactions to them. Patients report bullying, social exclusion, and misunderstandings about the degree of infection of the disease, which leads to social withdrawal and increased emotional distress. Sexual problems are also common and are associated with reduced self-esteem and shame. Results indicate that shame may contribute to symptom exacerbation via withdrawal and treatment non-compliance, though causal evidence is lacking.

The evidence found on disgust as a concomitant of dermatological diseases is less extensive than on shame. The results indicate, however, that self-disgust is an emotional response associated with dermatologic diseases, particularly in relation to visible skin rashes.

The second objective of the systematic review was to investigate the evidence for using compassion-based or mindfulness-based therapies to address self-disgust and shame in individuals with skin diseases. However, only three studies were found in total. The results on the effect of CFT-based therapy on shame and disgust in skin diseases are limited, but somewhat promising. At least there are already two RCT-studies that show that these interventions can reduce shame and skin discomfort in people with skin diseases.

The results show that further exploration of self-disgust and perceived disgust in dermatology is needed to better understand its impact on the psychological wellbeing of patients and to develop effective interventions to address this aspect of the patient experience. The relation of shame and self-disgust with fear and experience of social rejection, and the link with treatment non-compliance, support the assumption of the bio-psycho-social model that negative self-directed emotions increase stress and, as a result, skin disease symptoms. Additionally, a mediating role of shame and disgust in the links between skin disease and depression or lower quality of life indicates that negative emotions increase patients’ psychological burdens and may be associated with less effective coping. Longitudinal studies are necessary to test the causal role of shame and disgust in disease progression. Further studies should also focus strongly on the initial promising effects of CFT and MBT on shame and disgust in skin conditions.

While this systematic review provides valuable insights, several limitations should be considered. First, the overall quality of the included studies was moderate, with a particularly high risk of bias in many of the controlled intervention studies. Second, comparability across studies was limited due to substantial heterogeneity in both measurement instruments and the use of core constructs. As shown in Table 1, similar constructs such as “stigma,” “self-stigma,” and “self-disgust” were operationalized using more than 19 different instruments, reflecting inconsistent conceptualization across studies. Third, the number of intervention studies, especially randomized controlled trials, was low, which restricts the strength of conclusions regarding treatment effectiveness. Lastly, the wide publication span of the included studies (1982–2022) may have introduced cohort effects, potentially contributing to variability in findings.

**TABLE 1:** Details of included studies.

	Author, year, country	*n*	Design	Population	Main results	Constructs of interest	Measure
**Shame, embarrassment and self-stigma**							
50	Aberer et al. (2020), Austria	201	Cross-sectional survey	Psoriasis, eczema and other skin diseases	Patients with psoriasis, inflammatory skin disease or eczema had especially high levels of skin shame, but the patient groups did not differ in other aspects of shame.	Shame	SSS-24^1^, SHAME^2^
64	Almeida et al. (2020), Portugal	75	Cross-sectional survey	Psoriasis	Years of education, impact on social life and body image contribute to psoriasis disability. Body-image related cognitions play an important part as a moderator in the relation between symptom severity, acceptance and psoriasis disability.	Self-compassion, body image	CFQ-BI^3^, SCS^4^
34	Armstrong et al. (2012), USA	5,604	Cross-sectional survey	Psoriasis	Psoriasis and psoriatic arthritis affected overall emotional wellbeing in 88% of patients. Most patients reported embarrassment (87%), and self-consciousness (89%).	Embarrassment	Original
35	Augustin and Radtke (2014), Germany	n.a.	Review	Psoriasis	Patients with psoriasis suffer from various impairments including embarrassment and stigmatization.	Embarrassment, stigma	n.a.
62	Barisone et al. (2020), international	n.a.	Review	various skin diseases	Review of qualitative studies on sexuality and intimate relationships suggested among others embarrassment and shame as a core theme.	Embarrassment, shame	n.a.
71	Buckwalker, K.C. (1982), USA	–	Theoretical article	Psoriasis and other skin diseases	Points out impairments in sexuality due to shame and stigmatization among patients with psoriasis.	Shame, stigma	n.a.
51	Coates et al. (2020), intern.	1286	Global survey	Psoriatic arthritis	Social impacts included emotional distress (58%), social shame or disapproval (32%), and ceased participation in social activities (45%).	Shame	PsAID^5^
63	Fisher et al. (2020), Israel	20	Qualitative interviews	Psoriasis and hidradenitis suppurativa	Both diseases share similar experiences of shame.	Shame, embarrassment, self-disgust	Qualitative
73	George et al. (2021), UK	21	Qualitative study	Psoriasis	Patients reported impairment due to shame and fear of social exclusions as well as avoidance behavior.	Shame, stigma	DLQI^6^ Qualitative
39	Ginsburg and Link (1989), USA	100	Cross-sectional survey	Psoriasis	Six dimensions of stigma experience were identified: anticipation of rejection, feeling of being flawed, sensitivity to others’ attitudes, guilt and shame, secretiveness, and positive attitudes.	Shame, stigma, self-stigma, embarrassment	Original
38	Ginsburg and Link (1993), USA	100	Cross-sectional survey	Psoriasis	19% of patients experienced 50 episodes of gross rejection due to psoriasis Rejection can lead to feeling stigmatized and to increased alcohol consumption.	Stigma	Original
59	Gochnauer et al. (2017), USA	n.a.	Review	Atopic Eczema	Atopic eczema is among others accompanied with experiences of stigmatization and feelings of embarrassment. Different tools to assess different aspects of quality of life are portrayed.	Embarrassment, stigma	n.a.
54	Hayashi et al. (2014), Japan	210	Cross-sectional survey	Acne	Patients with acne experienced more severe emotional effects from their skin disease than functional or symptomatic effects. 75% reported feelings of embarrassment.	Embarrassment	Skindex-16^7^
57	Hazarika and Archana (2016), India	100	Cross-sectional survey	Acne	88% of patients reported embarrassment/self-consciousness due to acne Degree of embarrassment/self-consciousness showed statistically significant correlation to the severity of acne. Patients with facial acne reported feeling highly self-consciousness about their acne.	Embarrassment	DLQI^6^
48	Homayoon et al. (2020), Austria	132	Cross-sectional survey	Psoriasis	Higher levels of skin shame correlated with a greater disease burden, higher QoL, lower mental QoL. Higher levels of skin shame and less physical QoL in Patients compared to controls.	Shame	SSS-24^1^, SHAME^2^
68	Hrehorów et al. (2012), Poland	102	Cross-sectional survey	Psoriasis	Anticipation of rejection and feelings of guilt and shame were major aspects of stigmatization, the level of which correlated significantly with pruritus intensity, stress prior to exacerbation, depressive symptoms and quality of life.	Shame, stigma	StS^8^, FSQ^9^
49	Jafferany et al. (2018), USA	–	Review	Various skin diseases	Shame and embarrassment are common effects on patients with psoriasis, acne and eczema.	Shame, embarrassment	n.a.
52	Jankowiak et al. (2020), Poland	166	Cross-sectional survey	Psoriasis	Higher levels of shame are reported when there are visible skin lesions. Lower levels of shame in older patients.	Shame, stigma	FSQ^9^
7	Kellet and Gilbert (2010), UK	–	Review	Acne	Literature review that develops a biopsychosocial model for Acne illustrating the consequence of shame caused by acne under an evolutionary perspective.	Shame	n.a.
56	Kouris et al. (2017), international	–	Review	Psoriasis	Review pointing out embarrassment and shame are associated with and relevant consequences of psoriasis.	Embarrassment, shame, stigma	n.a.
33	Lahousen et al. (2016), Germany	342	Cross-sectional survey	Psoriasis	Patients with psoriasis reported higher levels of skin-related shame and disgust compared to skin-healthy controls.	Shame, self-disgust	Touch-Shame-Disgust-Questionnaire (TSD-Q)^10^
69	Magin et al. (2008), Australia	62	Qualitative study	Acne, atopic eczema, psoriasis	Teasing, taunting or bullying was a considerable problem for a significant minority of acne, psoriasis and atopic eczema participants.	Embarrassment, shame, stigma	Qualitative
17	Magin et al. (2009), Australia	29	Qualitative study	Psoriasis	Prominent sequelae of psoriasis were embarrassment, shame, impaired self-image, low self-esteem, self-consciousness and stigmatization. Psoriasis was associated with behavioral avoidance and effects on respondents’ sexuality.	Embarrassment, stigma	Qualitative
60	Ngaage and Agius (2018), international	–	Review	Various skin diseases incl. Acne	Review investigating the consequences of scares. Acne scars are associated with embarrassment.	Shame, embarrassment, self-stigma	n.a.
61	O’Neill et al. (2011), international	719	Cross-sectional survey	Psoriasis, Atopic Eczema	Psoriasis patients reported more embarrassment associated with itch than patients with Atopic eczema.	Embarrassment	n.r.
67	Ramsay and O’Reagan (1988), international	104	Cross-sectional survey	Psoriasis	The majority of patients reported feelings of embarrassment about their skin condition, cited non-sufferers’ beliefs about contagiosity as distressing, and referred to their bodies as “unclean.”	Stigma, embarrassment	Original
44	Russo et al. (2004), Australia	–	Review	Psoriasis	89% of psoriasis patients felt shame and embarrassment over their appearance.	Embarrassment, shame, stigma	n.a.
45	Rzepa et al. (2013), Poland	84	Cross-sectional survey	Psoriasis, Acne	30% of acne patients and 52% of psoriasis patients report shame because of the disease. psoriasis ranges in “top 10 of perceived embarrassing diseases.”	Shame	Original
46	Sampogna et al. (2012), Italy	936	Cross-sectional survey	Psoriasis	Around 35% of report shame “often” or “all the time”. Around 38% report embarrassment “often” or “all the time”; Higher Prevalence of Shame among women (OR: 1.6).	Shame	Skindex-29^11^
70	Schielein et al. (2020), Germany	344	Cross-sectional survey	Psoriasis	Most prevalent reason to avoid sexual activity is shame (*N* = 54 of 244 free text answers). Patients also report fear of rejection in sexual avoidance.	Shame	Original
65	Shah and Bewley (2014), UK	1	Case study	Psoriasis	Systemic therapeutic context and approach addressing feelings of shame cleared dermatological condition.	Shame	n.a.
47	Ständer et al. (2019), Germany	130	Interventional study	Psoriasis	125 of 130 subjects with psoriasis reported to be ashamed due to itchy skin, 127 reported to feel embarrassed and uncertain.	Shame, embarrassment	DLQI^6^ GerItchyQoL^12^
55	Tan et al. (2022a), international	723	Cross-sectional survey	Acne	Results show a positive correlation between embarrassment and acne severity. Patients also reported concerns with stigma, low self-esteem and avoidance of public exposure.	Shame, stigma, embarrassment	DLQI^6^ DCQ^13^ FASQoL^14^
58	Tan et al. (2022b), international	30	Qualitative study	Acne	27.5% of patients with acne scarring show embarrassment or self-consciousness and significant limitations in daily activities related to embarrassment due to acne scarring.	Shame, stigma, embarrassment	Qualitative
53	Torales et al. (2020), international	n.a.	Review	Psoriasis	Pathophysiology of psoriasis linked to maladaptive psychological characteristics like shame (i.a.) due to visible skin conditions and psychiatric disorders via inflammation (activation of HPA axis).	Shame	n.a.
72	Vladut and Kallay (2010), –	n.a.	Review	Psoriasis	Pointing out psychological burden of psoriasis: Shame in patients may be associated with problems in interpersonal and professional areas. Avoidance of social contact due to shame heightens probability of depression. Multimodal treatment is important.	Shame	n.a.
66	Wojciechowska-Zdrojowy et al. (2018), Poland	76	Cross-sectional survey	Psoriasis	Men with psoriasis reported feelings of embarrassment and diminished sense of attractiveness due to visible skin lesions, and shame with sexual partners, all of which was correlated with depression and low quality of life.	Shame	DLQI^6^, Original
**Self-disgust and experience of disgust in dermatological diseases**							
77	Kleyn et al. (2009), UK	26	Quasi-experimental study	Psoriasis	Study investigates neural responses in FMRI of psoriasis patients on disgusted faces compared to healthy controls. Patients showed smaller activation in bilateral insular cortex than controls. Furthermore, they showed reduced recognition of disgust intensity compared to controls. It suggested that this might be due to established coping mechanism to protect oneself from disgust reaction from others.	Disgust	fMRI
74	Mento et al. (2020), international	–	Review	Various skin diseases	Focussing on negative emotions in skin diseases. Conclusion is drawn that anger and disgust are neglected in studies.	Disgust	n.a.
76	Narayanan et al. (2015), international	50	Qualitative study	Psoriasis	Patients described themselves with disgust and self-loathing and report various social difficulties in everyday life.	Disgust, embarrassment, stigma	Qualitative
32	Schienle and Wabnegger (2022), Austria	193	Cross-sectional survey	Various skin diseases (incl. Psoriasis)	64% of patients showed elevated self-disgust with depression, stigmatization experience among others served as predictors for self-disgust. Depression mediated the relationship between stigmatization and self-disgust.	Disgust	QASD^15^
41	van Beugen et al. (2016), Netherlands	247	Cross-sectional survey	Psoriasis, alopecia	Patients with psoriasis and their significant others showed an increased behavioral avoidance bias of disgusted faces, which is absent in patients with alopecia and their SOs. Patients with alocepia and their SOs, but not psoriasis patients or SOs, show an attentional bias to disease-related stimuli.	Experience of disgust, self-stigma	Original
75	Wahl et al. (2002), Norway	22	Qualitative study	Psoriasis	Experience of rashes leads to embarrassment. Feelings of disgust with ones own body especially when rashes appear on hard to cover skin areas; Feelings of disgust with treatment procedures; own body perceived as “offensive.”	Disgust, embarrassment	Qualitative
	Psychotherapy for dermatological diseases: compassion-focused therapeutic approaches					Therapeutic approach	
78	Kelly et al. (2009), Canada	75	RCT	Acne	RCT on Compassion oriented interventions for depressed, distressed acne patients. Self-soothing intervention lowered shame and skin complaints. Attack-resisting interventions lowered depression, shame, and skin complaints, and was especially effective at lowering depression for self-critics.	CFT, imagery, shame, embarrassment	Original, ESS^16^, Skindex-16^7^
79	Krasuka et al. (2018), UK	5	Interventional study, no control group	Various skin diseases	4-week self-help programme, showing mixed results with a reduction in shame and self-criticism in a couple of patients and hightend scores with others	CFT, mindfulness, self-compassionate imagery, shame	OAS^17^, FSCRS^18^, DAS24^19^
**Psychotherapy for dermatological diseases: mindfulness-based therapeutic approaches**							
80	Muftin et al. (2022), UK	130	RCT	Psoriasis	Both a 4-week CFT online self-help as well as mindfulness-based self-help is acceptable and helpful to reduce shame and improve quality of life in patients with psoriasis.	Self-compassion, mindfulness, shame	OAS^17^, FSCRS^18^

^1^Skin Shame Scale (81).

^2^Shame Assessment Scale for multifarious expression of shame (82).

^3^Cognitive Fusion Questionnaire—body image (83).

^4^Self-Compassion Scale (84).

^5^Psoriatic Arthritis Impact of Disease questionnaire (85).

^6^Dermatology Life Quality Index (86).

^7^Skindex-16 (87).

^8^Stigmatization Scale (88).

^9^Feelings of Stigmatization Questionnaire (38).

^10^Touch-Shame-Disgust-Questionnaire (89).

^11^Skindex-29 (90).

^12^Pruritus-specific Life Quality Index—German version (91).

^13^Dysmorphic Concerns Questionnaire (92).

^14^Facial Acne Quality of Life Questionnaire (93).

^15^Questionnaire for the Assessment of Self-Disgust (94).

^16^Experiences of Shame Scale (95).

^17^Other as Shamer Scale (96).

^18^Forms of Self-Criticizing/Attacking and Self-Reassuring Scale (97).

^19^Derriford Appearance Scale 24 (98).

In conclusion, the findings of this review align with the adapted biopsychosocial model of skin disease progression. Patients with the three included inflammatory skin diseases are burdened by shame and self-disgust —central emotional responses to negative self-appraisal and social rejection—which are often related to experiences and fear of social rejection. These processes mirror the model’s pathways from skin conditions to negative self-related emotions, social difficulties, and increased arousal, though causal evidence is lacking. Mindfulness- and compassion-based approaches appear promising in addressing these shame and self-disgust and improving patient outcomes.

## Data Availability

The original contributions presented in this study are included in this article/Supplementary material, further inquiries can be directed to the corresponding author.
