# Supplementary material for: Shame and disgust in patients with inflammatory skin diseases: a systematic review of psychological correlates and psychotherapeutic approaches
Source: Front Med (Lausanne). 2025 Jun 30;12:1620940. doi: 10.3389/fmed.2025.1620940 (PMC12256482; doi:10.3389/fmed.2025.1620940)
Supplement: Supplementary file 1 [file Table_1.docx]

Supplement Table 1: Study quality assessment / Risk of Bias Assessment

| Systematic Reviews and Meta-Analyses | Is the review based on a focused question that is adequately formulated and described? | Were eligibility criteria for included and excluded studies predefined and specified? | Did the literature search strategy use a comprehensive, systematic approach? | Were titles, abstracts, and full-text articles dually and independently reviewed for inclusion and exclusion to minimize bias? | Was the quality of each included study rated independently by two or more reviewers using a standard method to appraise its internal validity? | Were the included studies listed along with important characteristics and results of each study? | Was publication bias assessed? | Was heterogeneity assessed? (This question applies only to meta-analyses.) | Summary Quality |  |  |  |  |  |  |
| --- | --- | --- | --- | --- | --- | --- | --- | --- | --- | --- | --- | --- | --- | --- | --- |
| Augustin & Radtke (2014), Germany | Yes | no | no | no | no | no | no | NA | poor |  |  |  |  |  |  |
| Barisone et al. (2020), international | yes | yes | yes | yes | yes | yes | yes | NA | good |  |  |  |  |  |  |
| Buckwalker, K.C. (1982), USA | yes | no | no | NR | NR | no | no | NA | poor |  |  |  |  |  |  |
| Gochnauer et al. (2017), USA | yes | no | no | NR | no | no | no | NA | poor |  |  |  |  |  |  |
| Jafferany et al. (2018), USA | yes | no | no | NR | no | no | no | NA | poor |  |  |  |  |  |  |
| Kellet & Gilbert (2010), UK | yes | no | no | no | no | no | no | NA | poor |  |  |  |  |  |  |
| Kouris et al. (2017), international | yes | no | no | no | no | no | no | NA | poor |  |  |  |  |  |  |
| Mento et al. (2020), international | yes | yes | yes | NR | NR | yes | no | NA | fair |  |  |  |  |  |  |
| Ngaage & Agius (2018), international | yes | no | no | no | no | no | no | NA | poor |  |  |  |  |  |  |
| Russo et al. (2004), Australia | yes | no | no | no | no | no | no | NA | poor |  |  |  |  |  |  |
| Torales et al. (2020), international | no | no | no | no | no | no | no | NA | poor |  |  |  |  |  |  |
| Vladut & Kallay (2010), - | yes | no | no | no | no | no | no | NA | poor |  |  |  |  |  |  |
| Controlled Intervention Studies | Was the study described as randomized, a randomized trial, a randomized clinical trial, or an RCT? | Was the method of randomization adequate (i.e., use of randomly generated assignment)? | Was the treatment allocation concealed (so that assignments could not be predicted)? | Were study participants and providers blinded to treatment group assignment? | Were the people assessing the outcomes blinded to the participants' group assignments? | Were the groups similar at baseline on important characteristics that could affect outcomes (e.g., demographics, risk factors, co-morbid conditions)? | Was the overall drop-out rate from the study at endpoint 20% or lower of the number allocated to treatment? | Was the differential drop-out rate (between treatment groups) at endpoint 15 percentage points or lower? | Was there high adherence to the intervention protocols for each treatment group? | Were other interventions avoided or similar in the groups (e.g., similar background treatments)? | Were outcomes assessed using valid and reliable measures, implemented consistently across all study participants? | Did the authors report that the sample size was sufficiently large to be able to detect a difference in the main outcome between groups with at least 80% power? | Were outcomes reported or subgroups analyzed prespecified (i.e., identified before analyses were conducted)? | Were all randomized participants analyzed in the group to which they were originally assigned, i.e., did they use an intention-to-treat analysis? | Summary Quality |
| Kelly et al. (2009), Canada | yes | NR | NR | No | NR | Yes | Yes | Yes | NA | Yes | Yes | No | No | Yes | fair |
| Muftin et al. (2022), UK | yes | Yes | No | Yes | NA | no | No | No | NR | NR | Yes | No | NR | Yes | fair |
| Observational Cohort and Cross-Sectional Studies | Was the research question or objective in this paper clearly stated? | Was the study population clearly specified and defined? | Was the participation rate of eligible persons at least 50%? | Were all the subjects selected or recruited from the same or similar populations? | Was a sample size justification, power description, or variance and effect estimates provided | For the analyses in this paper, were the exposure(s) of interest measured prior to the outcome(s) being measured? | Was the timeframe sufficient so that one could reasonably expect to see an association between exposure and outcome if it existed? | For exposures that can vary in amount or level, did the study examine different levels of the exposure? | Were the exposure measures (independent variables) clearly defined, valid, reliable, and implemented consistently across all study participants? | Was the exposure(s) assessed more than once over time? | Were the outcome measures (dependent variables) clearly defined, valid, reliable, and implemented? Consistently across all study participants? | Were the outcome assessors blinded to the exposure status of participants? | Was loss to follow-up after baseline 20% or less? | Were key potential confounding variables measured and adjusted statistically for their impact on the relationship? between exposure(s) and outcome(s)? | Summary Quality |
| Aberer et al. (2020), Austria | yes | yes | yes | yes | no | no | no | no | yes | no | yes | yes | NA | yes | fair |
| Almeida et al. (2020), Portugal | yes | yes | NR | yes | yes | no | no | yes | yes | no | yes | yes | NA | yes | fair |
| Armstrong et al. (2012), USA | yes | yes | NA | CD | no | no | no | yes | yes | no | yes | yes | NR | NA | fair |
| Coates et al. (2020), international | yes | yes | yes | yes | no | no | no | yes | yes | no | yes | yes | NA | NA | fair |
| Ginsburg & Link (1993), USA | yes | yes | yes | yes | no | no | no | yes | yes | no | no | NA | NA | yes | fair |
| Hayashi et al. (2014), Japan | yes | yes | NR | no | no | no | no | yes | yes | no | yes | yes | NA | no | fair |
| Hazarika & Archana (2016), India | yes | yes | NR | yes | no | no | no | yes | yes | no | yes | yes | NA | yes | fair |
| Homayoon et al. (2020), Austria | yes | yes | NR | yes | yes | no | no | yes | yes | no | yes | yes | NA | yes | fair |
| Hrehorów et al. (2011), Poland | yes | yes | NR | NR | no | no | no | yes | yes | no | yes | yes | NA | no | fair |
| Jankowiak et al. (2020), Poland | yes | yes | NR | yes | no | yes | yes | NA | yes | no | yes | NA | NA | no | good |
| Kleyn et al. (2009), UK | yes | yes | NR | yes | no | yes | yes | yes | yes | no | yes | NA | NA | no | fair |
| Krasuka et al. (2018), UK | yes | yes | no | yes | no | yes | yes | no | NR | no | yes | NA | no | NR | poor |
| Lahousen et al. (2016), Germany | yes | yes | yes | yes | no | no | no | yes | yes | no | yes | NA | NA | yes | good |
| O’Neill et al. (2011), international | yes | yes | NR | yes | no | yes | yes | no | yes | no | yes | NA | NA | no | fair |
| Ramsay & O’Reagan (1988), international | no | yes | NR | yes | no | no | no | yes | yes | no | yes | NA | NA | no | poor |
| Rzepa et al. (2013), Poland | yes | yes | yes | yes | no | yes | yes | no | NR | no | NR | NA | NA | no | poor |
| Sampogna et al. (2012), Italy | yes | yes | yes | yes | no | yes | yes | yes | yes | no | yes | NA | NA | no | good |
| Schielein et al. (2020), Germany | yes | yes | NA | yes | no | yes | yes | yes | no | no | no | NA | NA | no | fair |
| Schienle & Wabnegger (2022), Austria | yes | yes | NR | yes | yes | yes | yes | no | NR | no | yes | NA | NA | no | fair |
| Shah & Bewley (2014), UK | yes | NA | NA | NA | NA | NA | NA | NA | NA | NA | NA | NA | NA | NA | poor |
| Ständer et al. (2019), Germany | yes | yes | NR | yes | no | yes | yes | yes | yes | no | yes | NA | NA | no | good |
| Tan et al. (2022a), international | yes | yes | no | yes | no | yes | yes | yes | yes | no | yes | NA | NA | no | good |
| van Beugen et al. (2016), Netherlands | yes | yes | yes | yes | no | no | no | yes | yes | no | yes | NA | NA | yes | good |
| Wojciechowska-Zdrojowy et al. (2018), Poland | yes | no | NR | yes | no | no | no | yes | yes | no | yes | NA | NA | yes | fair |
| Qualitative studies | Domain 1a) Research team and reflexivity (x/5) | Domain 1b) Relationship with participants (x/3) | Domain 2a) Theoretical framework (x/1) | Domain 2b) Participant Selection (x/4) | Domain 2c) Setting (x/3) | Domain 2d) Data colllection (x/7) | Domain 3a) Data Analysis (x/5) | Domain 3b) Reporting (x/4) | Total Points reported (x/32) | Summary Quality |  |  |  |  |  |
| Fisher et al. (2020), Israel | 3/5 | 1/3 | 1/1 | 4/4 | 3/3 | 3/7 | 2/5 | 3/4 | 20/32 | good |  |  |  |  |  |
| George et al. (2021), UK | 2/5 | 2/3 | 1/1 | 2/4 | 1/3 | 1/7 | 4/5 | 4/4 | 17/32 | fair |  |  |  |  |  |
| Magin et al. (2008), Australia | 5/5 | 0/3 | 1/1 | 2/4 | 1/3 | 2/7 | 2/5 | 4/4 | 17/32 | fair |  |  |  |  |  |
| Magin et al. (2009), Australia | 5/5 | 0/3 | 1/1 | 3/4 | 1/3 | 4/8 | 2/5 | 4/4 | 20/32 | good |  |  |  |  |  |
| Narayanan et al. (2014), international | 3/5 | 0/3 | 1/1 | 3/4 | 3/3 | 3/8 | 1/5 | 4/4 | 18/32 | fair |  |  |  |  |  |
| Tan et al. (2022b), international | 2/5 | 0/3 | 1/3 | 3/4 | 3/3 | 2/8 | 2/5 | 3/4 | 16/32 | fair |  |  |  |  |  |
| Wahl et al. (2002), Norway | 0/5 | 0/3 | 1/1 | 3/4 | 2/3 | 3/8 | 1/5 | 4/4 | 14/32 | fair |  |  |  |  |  |

CD = cannot determine, NA = not applicable, NR = not reported, N/A = no answer
